# Applications of Artificial Intelligence, Machine Learning, and Deep Learning in Nutrition: A Systematic Review

**DOI:** 10.3390/nu16071073

**Published:** 2024-04-06

**Authors:** Tagne Poupi Theodore Armand, Kintoh Allen Nfor, Jung-In Kim, Hee-Cheol Kim

**Affiliations:** 1 Institute of Digital Anti-Aging Healthcare, Inje University, Gimhae 50834, Republic of Korea; poupiarmand2@gmail.com (T.P.T.A.); fdsnkiji@inje.ac.kr (J.-I.K.); 2 Department of Computer Engineering, Inje University, Gimhae 50834, Republic of Korea; nforallen94@yahoo.com; 3 College of AI Convergence, u-AHRC, Inje University, Gimhae 50834, Republic of Korea

**Keywords:** artificial intelligence, machine learning, deep learning, nutrition, diet

## Abstract

In industry 4.0, where the automation and digitalization of entities and processes are fundamental, artificial intelligence (AI) is increasingly becoming a pivotal tool offering innovative solutions in various domains. In this context, nutrition, a critical aspect of public health, is no exception to the fields influenced by the integration of AI technology. This study aims to comprehensively investigate the current landscape of AI in nutrition, providing a deep understanding of the potential of AI, machine learning (ML), and deep learning (DL) in nutrition sciences and highlighting eventual challenges and futuristic directions. A hybrid approach from the systematic literature review (SLR) guidelines and the preferred reporting items for systematic reviews and meta-analyses (PRISMA) guidelines was adopted to systematically analyze the scientific literature from a search of major databases on artificial intelligence in nutrition sciences. A rigorous study selection was conducted using the most appropriate eligibility criteria, followed by a methodological quality assessment ensuring the robustness of the included studies. This review identifies several AI applications in nutrition, spanning smart and personalized nutrition, dietary assessment, food recognition and tracking, predictive modeling for disease prevention, and disease diagnosis and monitoring. The selected studies demonstrated the versatility of machine learning and deep learning techniques in handling complex relationships within nutritional datasets. This study provides a comprehensive overview of the current state of AI applications in nutrition sciences and identifies challenges and opportunities. With the rapid advancement in AI, its integration into nutrition holds significant promise to enhance individual nutritional outcomes and optimize dietary recommendations. Researchers, policymakers, and healthcare professionals can utilize this research to design future projects and support evidence-based decision-making in AI for nutrition and dietary guidance.

## 1. Introduction

Nutrition is a complex field that explores the connection between diet, health, and disease [1]. At its heart, it concerns the physiological and chemical processes involved in nourishment and how food substances provide energy or are converted into body tissues [2]. These processes are crucial to life, affecting overall health, physical development, and disease prevention. The significance of nutrition is not exaggerated. It provides information about how dietary decisions affect overall health and well-being. Melaku et al. [3] state that the global burden of non-communicable diseases, many of which are diet-related, is significant in public health. Nutrition provides the principles upon which to understand these issues and develop solutions. Additionally, nutrition is important for understanding the prevalence of various diseases. According to Popkin et al. [4], global dietary shifts are associated with changes in disease patterns. This shift highlights the value of nutrition in addressing global health concerns like obesity, diabetes, and heart disease [5].

Besides preventing disease, nutrition has a significant role in treating and managing various health issues [6]. According to a report from the American Diabetes Association, 2020, medical nutrition therapy, which is integral to dealing with diseases like diabetes, is based on the principles of nutrition science [7]. Furthermore, the field is growing rapidly due to the combination of genomics and personalized nutrition. Personalized nutrition uses individual genetic information to recommend dietary habits [8]; this could significantly impact disease prevention and health improvement.

The field of nutrition has traditionally relied on observational studies and clinical trials, but the advent of AI has supplanted this. These technologies, including machine learning, deep learning, and data analysis, have the potential to uncover complex relationships in large datasets, identify patterns, and generate actionable information [9,10]. From dietary personalization to preventative models that predict disease, the potential uses of AI in nutrition are numerous and widespread. Integrating AI applications in nutrition facilitates technological advancements reshaping the landscape of dietary interventions [11]. AI techniques hold immense promise in this data-driven era for revolutionizing how we understand, monitor, and optimize nutritional outcomes.

In nutrition, AI can be defined as applying conceptual algorithms, machine learning, and deep learning techniques to analyze, interpret, and make informed decisions from various datasets related to nutritional data, dietary patterns, and other health factors [11,12,13]. Leveraging machine learning algorithms, random forests can be used to analyze genetics and dietary data to understand how nutrients influence human genetic variations [14,15]. Moreover, collaborative filtering techniques are widely used in personalized nutrition recommendations, while deep learning methods such as convolutional neural networks (CNN) and transfer learning pre-trained models (Resnet, EfficientNet, etc.) assist in identifying and classifying meals using food images to detect dietary patterns further to assess nutritional content [16,17,18].

This study gathers and synthesizes existing evidence by providing a comprehensive overview of how AI techniques are harnessed to address nutritional challenges. The integration of AI into nutrition science is rapidly growing, and understanding the current landscape is fundamental for researchers and policymakers. Therefore, the authors conducted a systematic literature review on artificial intelligence and its sub-fields from 2019 to 2024. Studies were systematically collected, appraised, and synthesized with emphasis on applications of AI techniques in nutrition, challenges, and limitations. 

The paper is organized as follows: Section 2 describes the adopted methodology for conducting the study, detailing the steps involved in the systematic literature review. Section 3 covers a descriptive analysis and content analysis of the selected research. In Section 4, the authors proposed a conceptual framework for the applications of AI in nutrition. Furthermore, the outcomes of this study are discussed in Section 5. Limitations are discussed in Section 6, followed by future directions, the identification of research gaps, and a conclusion. 

## 2. Methodology

A systematic literature review was conducted in this study regarding the applications of AI, machine learning, and deep learning techniques applied in nutrition sciences. A hybrid approach based on the (1) preferred reporting items for systematic reviews and meta-analysis (PRISMA) by Moher et al. [19] and (2) the systematic literature review (SLR) guidelines suggested by Manuel et al. [20] was used. The approach was structured and categorized into the following parts:

- Defining research question and objectives;
- Defining research scope;
- Literature selection;
- Validation of selected literature.

### 2.1. Research Question and Objectives

This systematic review aimed to identify and analyze the current state of artificial intelligence techniques applied in nutrition sciences. The following research question was formulated to guide our exploration: How have AI, ML, and deep learning been employed to impact and enhance our understanding, monitoring, and optimization of nutritional outcomes? The following objectives were set to answer the research question:

- Systematically review the literature: Conduct a comprehensive review of the existing literature on the applications of artificial intelligence in nutrition and identify studies that use artificial intelligence technologies such as ML and DL for various purposes in this field;
- Categorize the AI techniques: Analyze and classify the types of AI techniques implemented in nutritional research, including, but not limited to, ML and DL;
- Evaluate the methodological quality: Thoroughly assess the quality and methodological rigor of selected studies using predefined criteria to ensure the robustness of the assessment;
- Identify challenges and limitations and proposed future directions: Identify the challenges associated with integrating AI technology into nutrition, highlighting potential areas for improvement and future research based on findings.

### 2.2. Defining the Research Scope

This study was carried out within AI applications in nutrition science. The sub-domains of AI are numerous and needed to be defined for this study. In this study, the authors framed the research by defining specific AI sections to be considered. Therefore, the following areas were adopted: machine learning and deep learning techniques. 

### 2.3. Literature Selection Process

**Figure 1:**
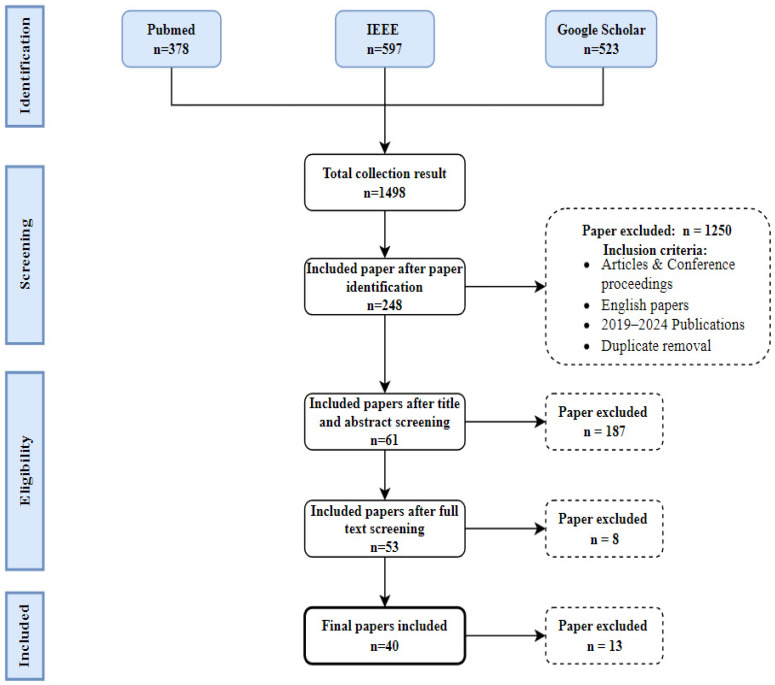
The literature selection process.

A systematic review requires details of the search criteria, search terms, databases, publication period, and inclusion and exclusion criteria used in the research selection process. By searching electronic databases that provide articles on artificial intelligence and nutrition sciences, the authors concluded that PubMed, IEEE, and Google Scholar have the highest number of articles relevant to this study as they contain the most suitable scientific publications within their scope of study. An additional search of other databases did not significantly affect the search results; therefore, the databases above were adopted. The selection process is illustrated in Figure 1 below. 

Firstly, the authors identified the relevant literature for artificial intelligence (machine learning, deep learning) and nutrition (diet, food, dietary assessment, personalized nutrition, meal planning, healthy eating, etc.), resulting in 1498 research items. The authors included original research articles and conference proceedings, and the publication language was restricted to English. This first approach was used to gain insight into the current research state. The authors, therefore, limited the timeframe used in this research study to five years, from January 2019 to January 2024. Duplicate papers were excluded from the resulting papers, for which 248 research items were included from the initial dataset used in this study. The identification process can be summed in a final meta-search query formulated as follows:

(TITLE-ABS-KEY (“artificial intelligence” OR “machine learning” OR “deep learning” OR “ recommendation”) AND TITLE-ABS-KEY (“diet” OR “nutrition” OR “food” OR “dietary assessment” OR “personalized*” OR “meal planning” OR “healthy eating”)) AND (LIMIT-TO (DOCTYPE, “cp”) OR LIMIT-TO (DOCTYPE, “ar”) OR LIMIT-TO (DOCTYPE, “cr”)) AND (LIMIT-TO (SUBJAREA, “ENGI”)) AND (LIMIT-TO (PUBYEAR, 2024) OR LIMIT-TO (PUBYEAR, 2023) OR LIMIT-TO (PUBYEAR, 2022) OR LIMIT-TO (PUBYEAR, 2021) OR LIMIT-TO (PUBYEAR, 2020) OR LIMIT-TO (PUBYEAR, 2019)) AND (LIMIT-TO (LANGUAGE, “English”)). 

Moreover, this meta-search query was validated using the computed ranking of all the keywords identified in this study. Table 1 shows the characteristics of the search string. 

The second step of the selection process is the title and abstract screening. To prevent bias in the selection process, two independent researchers screened the titles and abstracts of articles based on this study’s predefined objectives and scope. Since the included researchers approved AI-based methods, the selection was based on studies that intersect with diet, nutrition, dietary assessment, personalized nutrition, food, meal planning, healthy eating, etc. This phase resulted in the inclusion of 61 research items. After screening the titles and abstracts, the researchers continued the full-text screening process. The screening was deeper as the researchers had to ensure the selection matched this study’s objectives. The exclusion and inclusion criteria were used to guide the choice of research items based on their full text, and the researchers discussed them until a consensus was reached on whether to exclude or include a study in this study. This step resulted in 53 selected papers relevant to the domain of artificial intelligence and nutrition. Within this area, 40 studies were categorized as relatively suitable for this study. 

### 2.4. Validation of the Selected Literature

**Figure 2:**
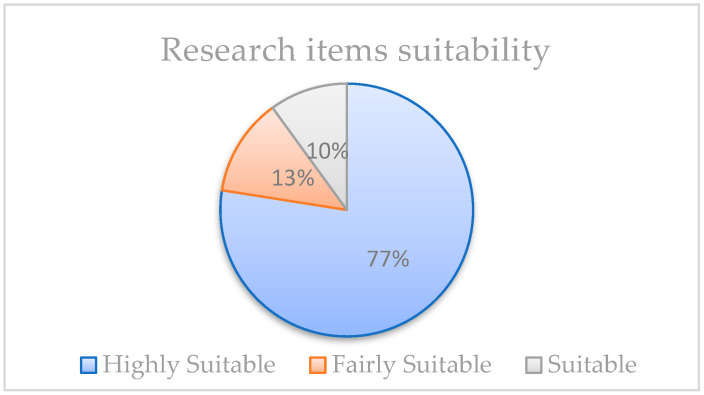
Validated studies are included in the systematic review.

To ensure the quality of the included literature, the authors used the quality criteria defined in the process described by [19,20]. The quality assessment was performed by coding the selected studies and assigning scores based on established validity, reliability, objectivity, and generalizability criteria. The code scores were as follows: “1” (indicating highly suitable), “2” (indicating fairly suitable), and “3” (indicating suitable). The authors further assessed reliability by evaluating notable differences in the scorings. Papers with no significant difference scored lower (2 or 3) and were excluded from the research process. The research team re-evaluated papers with substantial differences to identify unambiguous research results. From an AI and nutrition perspective, considering our research objectives, 31 research items were evaluated as highly appropriate for this study among the 40 shortlisted. Figure 2 below shows the proportion of highly suitable studies for selection validation.

## 3. Results

This section examines the descriptive findings obtained from the selected studies. Additionally, the chosen study’s content is analyzed and discussed in clusters.

### 3.1. Descriptive Analysis

Initially, 1498 papers related to the application of artificial intelligence in nutrition were identified, and a subset of 40 papers was specially earmarked for the domain of artificial intelligence in nutrition. These papers were recognized as relevant, and the authors deemed them appropriate for subsequent analysis in the present research study. Table 2 shows the proportionality of the selected records according to their suitability for the study.

Out of the 40 full texts in the artificial intelligence and nutrition domain, 31 papers (77.5%) were classified as highly suitable papers, 5 papers (12.5%) were identified as fairly suitable, and 4 papers (10%) were identified as suitable vis-à-vis the aim of this research study. The authors, therefore, decided to work with the 31 highly suitable papers for the rest of the study.

**Figure 3:**
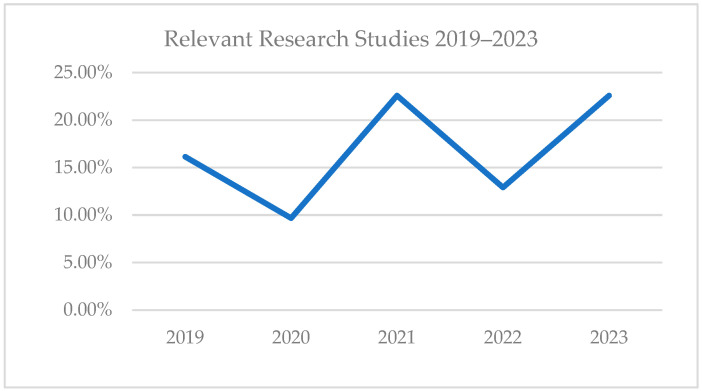
Evolution of relevant research studies in 2019–2024.

Among the 31 papers finally included in this study, five papers (16.13%) were published in 2019, three papers (9.68%) were published in 2020, seven papers (22.58%) were published in 2021, four papers (12.90%) were published in 2022, and seven papers (22.58%) published in 2023 as shown in Figure 3 below. In the chosen period (2019–2024), though we can observe a fluctuation in the number of publications in the selected set, the highest set of papers involved in this review was published in 2023, thus signifying the recent interest in this research area.

**Figure 4:**
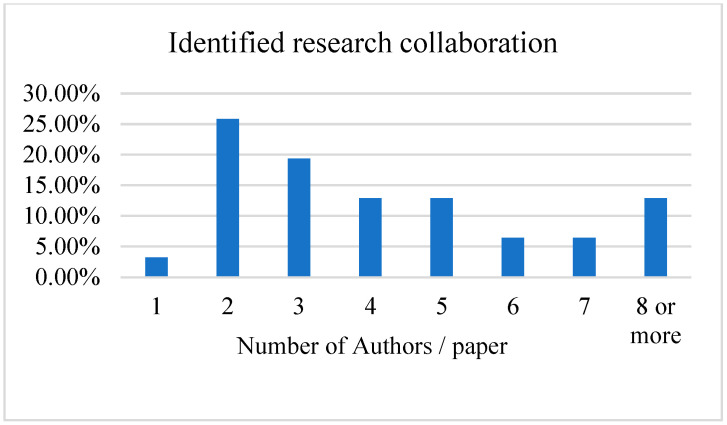
Overview of identified research collaboration.

Figure 4 shows an overview of identified research collaboration studies based on the systematic literature review.

Based on our selection, one paper (3.23%) was published by one author, eight papers (25.81%) were published by two authors, six papers (19.35%) were published by three authors, four papers (12.90%) were published by four authors, four papers (12.90%) were published by five authors, two papers (6.45%) were published by six authors, two papers (6.45%) were published by seven authors, and four (12.90%) papers were published by more than eight authors. This signifies that most studies in AI and nutrition are a product of collaboration, certainly among researchers from different research backgrounds, as shown below. 

**Figure 5:**
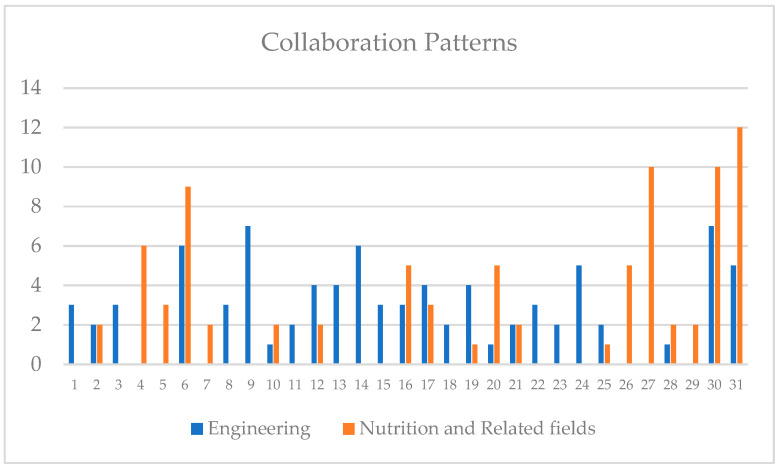
Collaboration patterns between engineering and nutrition/related authors.

We investigated the research areas of the authors of the selected papers. The authors were classified into two sets based on their affiliations: (1) engineering for those from computing and AI-related backgrounds and (2) those from a nutrition-related background. Figure 5 below shows the collaboration patterns between the author’s research areas across the 31 selected papers. Though a few papers are written by authors from one research area, most papers result from a research collaboration between engineers and nutritionists. This can be justified because designing a fully functional system for a real-life setting requires high-level knowledge from experts from both backgrounds. 

**Figure 6:**
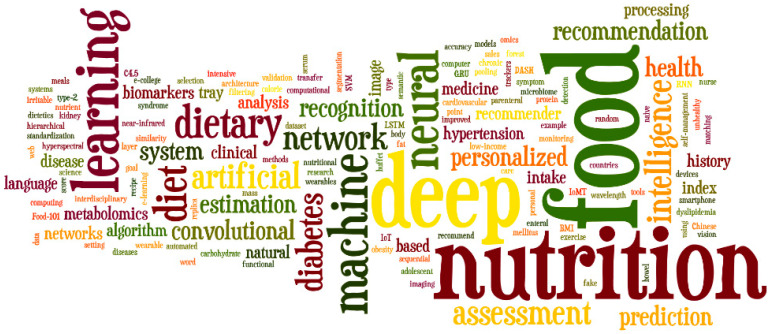
Keyword analysis.

The authors further extracted the keywords from the 31 final papers used and performed a keyword analysis. The results are shown in Figure 6.

The keywords were analyzed using Wordle software [21]. Therefore, the size of the words and letters is directly proportional to the frequency of the word from the final sample of keywords from 31 papers used in this study. As expected, the most important keywords are “Nutrition”, “Food”, “Dietary”, “Deep Learning”, “Machine Learning”, “Recommendation”, “Artificial Intelligence”, “Recognition”, “Assessment”, and “Personalized”.

### 3.2. Content Analysis

In this section, the authors conducted an in-depth analysis of the full text of the selected papers. The following table presents a comprehensive overview of the systematic literature review, summarizing clusters, main references, records count, and the primary content of each identified study used.

The results regarding the application of artificial intelligence, machine learning, and deep learning in nutrition are classified into five main clusters and shown in Table 3. In subsequent paragraphs, we will elaborate on and summarize the content of the identified studies under their specified clusters.

#### 3.2.1. Smart and Personalized Nutrition

A significant area of application for artificial intelligence in nutrition is the development of smart and personalized dietary recommendations. Studies included in this systematic review have demonstrated the utilization of machine learning algorithms to analyze individual dietary patterns, health metrics, and genetic information to tailor dietary advice. These applications aim to enhance adherence to dietary guidelines and improve overall nutritional outcomes. A total of 10 (32.3%) papers were classified under the cluster “smart and personalized nutrition”. This cluster comprises different approaches using advanced technologies for personalized nutrition to enhance health outcomes. Kirk et al. [22] provides a comprehensive guide to machine learning in nutrition research by distinguishing machine learning approaches and giving examples of its application in precision nutrition and metabolomics. On the other hand, Zhu and Wang [23] emphasize the global nutritional security problem, discussing AI applications in personalized nutrition recommendations and providing examples such as predicting cooking parameters’ correlation with nutritional quality. Van Erp et al. [24] address the food challenge by providing the benefits of natural language processing and AI in recipe analysis for personalized recommendations. Based on purchase history data, Honda and Nishi [25] proposed a system to support healthy diet choices, including a household nutrition analysis and food recommendation system. Santhuja et al. [26] also suggested an intelligent, personalized nutrition guidance system utilizing IoT, ML algorithms, and image processing for real-time customized nutrition recommendations. Maurya et al. [27] focus on automating chronic kidney disease classification by providing customized diet plans. Mogaveera et al. [28] introduced a system designed to improve the health conditions of patients suffering from chronic diseases through personalized diet and exercise plans. Iwendi et al. [29] developed a recommendation system for patients and dieticians to tailor dietary choices based on individual health needs and preferences. Sookrah et al. [30] also proposed a DASH diet recommender system focusing on hypertensive patients using machine learning and content-based filtering. Finally, Iheanacho and Vincent [31] presented a system that utilizes deep learning, specifically convolutional neural networks (CNN) to classify and recommend healthy food recipes based on local dietary habits in West Africa. The collective findings of these research papers show the potential of integrating artificial intelligence techniques into nutrition and research for personalized and context-aware solutions to improve diet choices and healthy eating.

#### 3.2.2. Dietary Assessment

The assessment of nutrient intake is a fundamental aspect of nutrition research, and AI techniques contribute to enhancing the precision and efficiency of this process. Studies in this review have explored the use of AI for automating nutrient analysis from dietary records, food images, and other sources. This application not only streamlines data collection but also addresses challenges related to self-reporting biases. A total of six (19.4%) identified papers were classified under the cluster “Dietary Assessment”. Mezgec et al. [32] combine deep learning and natural language processing to improve automated dietary assessment. The authors tested the combination of an established food-choice research method (the “fake food buffet”) with a new technology to automate data collection and analysis. Their study has applications in nutrition and dietary assessment, as it provides a more efficient and accurate way to collect and analyze data, reducing the effort and biases introduced by manual handling. Folson et al. [33] conducted research to validate a mobile AI dietary assessment application called FRANI in Ghana. The authors involved 36 adolescent females aged 12–18 and compared the nutrient intake measured via FRANI with weighed records and 24-h recalls. Their results showed equal nutrient intake between FRANI and the other assessment methods. Moreover, this study demonstrates the possibilities of mobile AI technology in improving dietary assessment in low- and middle-income countries. Shi et al. [34] showed the use of deep learning to present a dietary automatic evaluation for Chinese tray meals. The authors further created and published the first Chinese tray meal dataset, ChinaLunchTray-99, containing 1185 tray meal images with 99 dish categories, enabling the development of a framework for automatic dietary assessment, including tray meal detection and nutrition estimation. Lu et al. [35] also developed an AI system called goFOODTM that can estimate a meal’s calorie and macronutrient content based on food images captured by a smartphone. The authors validated the system on two databases, which showed promising results compared to those of experienced dietitians. Their proposed system uses deep neural networks for food detection, segmentation, and recognition and a 3D reconstruction algorithm to estimate the volume of food intake. The goFOODTM system provides a simple and efficient solution for dietary assessment. Lastly, Lo et al. [36] propose a system that uses deep learning view synthesis techniques to estimate a person’s food intake from a single camera view of their plate. Van Asbroeck and Matthys [37] comprehensively compared various food image recognition platforms for dietary assessment. The authors evaluate the performances of different image recognition platforms in identifying types and amounts of food in images, aiming for consumer use. 

#### 3.2.3. Food Recognition and Tracking

Computer vision and image analysis advancements have enabled the automatic recognition and categorization of food items from images. The included studies demonstrate the application of deep learning models to analyze food images, providing a valuable tool for dietary assessment. This technology has implications for both research and interventions to improve nutritional habits. The authors assigned four (12.9%) identified papers to the “Food Recognition and Tracking” cluster. In this context, Li et al. [38] introduced an approach for estimating food nutrition using near-infrared hyperspectral imaging and deep learning techniques. The authors constructed a near-infrared spectral dataset of scrambled eggs with tomatoes. They proposed a deep learning method called OptmWave, which integrates two neural networks to predict protein content and select wavelengths simultaneously. Their study shows the potential of deep learning-based near-infrared hyperspectral imaging to predict nutrient content in food, thereby offering a new approach to food quality control and nutrition monitoring. Siemon et al. [39] provided a sequential transfer learning method using hierarchical clustering to improve the performance of deep learning-based food segmentation. Furthermore, they tested for segmenting foods in Danish school children’s meals for dietary intake monitoring. Their study provides a comprehensive framework for the automatic dietary assessment of Chinese tray meals, which addresses the challenges of tray meal detection and nutrition estimation using deep learning techniques. Their findings have practical significance for nutrition estimation and the statistical analysis of tray meals. Sripada et al. [40] developed deep learning algorithms and machine learning techniques to classify food images for nutritional assessment automatically. Their proposed approach combines a deep learning convolutional neural network (CNN) with a support vector machine (SVM) for food recognition and segmentation into healthy and unhealthy classes. Their experimental results show the effectiveness of the hybrid model, achieving an impressive accuracy rate of 97%, outperforming the standalone CNN model. The study shows the potential of AI-driven methods to enhance nutritional assessment and promote healthier eating practices. Limketkai et al. [41] discuss the potential for digital technology, particularly machine learning and mobile applications, to reshape the field of clinical nutrition by enabling food recognition and tracking, personalized interventions, real-time monitoring, and improved predictive capabilities.

#### 3.2.4. Predictive Modeling for Disease

Artificial intelligence is pivotal in predictive modeling for disease prevention within nutrition science. The included studies showcase the development of predictive models leveraging machine learning techniques to identify patterns associated with disease risk. These models often incorporate dietary data, lifestyle factors, and health indicators to forecast the likelihood of specific health outcomes. For the cluster “Predictive Modeling for Disease”, eight (25.8%) identified papers were assigned to this cluster. Salinari et al. [42] showed the potential of AI to improve the treatment of diseases; predict the diseases, care and medication of patients; and monitor patients in real time. They further describe the potential benefits of integrating these technologies into healthcare practices, including faster diagnosis, personalized medicine, disease evaluation and monitoring, and reduced healthcare costs. The backbone of their study suggests that digital technologies and AI are promising tools for health promotion, disease prevention, and management in nutrition assessment. Singer et al. [43] discuss the potential of artificial intelligence (AI) in clinical nutrition, focusing on how AI can be used to enhance screening and assessment, successful applications for identifying malnourished cancer patients and predicting clinical events in intensive care, and the ethical considerations and limitations associated with AI in clinical nutrition. Kim et al. [44] focused on the relationship between nutritional intake and the risk of developing overweight/obesity, dyslipidemia, hypertension, and type 2 diabetes mellitus. Mitchell et al. [45] proposed a personalized nutrition recommendation system, GlucoGoalie, to assist individuals in improving their nutrition and managing diabetes. They further demonstrate the potential of personalized nutrition recommendation systems, such as GlucoGoalie, to support individuals in managing their nutrition and health. Ma et al. [46] developed a deep learning algorithm to predict serum pyridoxal 5′-phosphate (PLP) concentration based on dietary intake and potential predictors. The authors provide valuable details of the relationships between dietary patterns and serum PLP, emphasizing the possibilities of artificial intelligence in nutrition assessment and disease prevention. Bond et al. [47] provide personalized interventions, assisting disease prevention and management and addressing ethical and regulatory concerns. Bhat and Ansari [48] were able to create machine learning techniques to predict diabetes and recommend proper diets for diabetic patients. The authors emphasize the importance of data analysis in healthcare, and they were able to present a model for diabetic prediction and diet recommendation. Finally, Veerasekhar Reddy et al. [49] propose an e-college nurse system focusing on BMI measurements and the early detection of Type-2 diabetes.

#### 3.2.5. Disease Diagnosis and Monitoring

Artificial intelligence extends its influence to the diagnosis and monitoring of nutrition-related diseases. Included studies illustrate the development of diagnostic models for conditions such as malnutrition, obesity, and metabolic disorders. These models often integrate diverse data sources, including clinical parameters, dietary information, and biomarkers. A total of three (9.7%) of the identified papers were assigned to the cluster “Disease Diagnosis and Monitoring”. Panagoulias et al. [50] focus on applying machine learning to personalized nutrition and health optimization biomarkers in this context. The authors present the use of metabolomics to study unique chemical fingerprints left by cellular processes and the potential of neural networks to evaluate nutritional biomarkers, predict body mass index (BMI), and discover dietary patterns. They propose a deep neural network model that classifies BMI based on a biochemistry profile and another model that classifies nutritional profiles. They also propose a biomarker-based deep learning system for personalized nutrition that predicts individual ideal body weight by classifying the body types into three categories, namely underweight, normal, and obese/overweight, in another study [51]. They further discuss the importance of biomarkers and metabolites for predicting health outcomes and personalizing nutrition. Karakan et al. [52] investigated the effect of an AI-based personalized diet on irritable bowel syndrome (IBS) patients. The authors enrolled 34 IBS-M patients and used an algorithm to optimize personalized nutrition strategies based on individual gut microbiome features. Their personalized nutrition intervention significantly improved the IBS Symptom Severity Score (IBS-SSS) and microbiome-derived IBS scores compared to the standard IBS diet.

The following Table 4 provides a comprehensive summary of the included studies. 

## 4. Conceptual Framework for Applying AI, ML, and DL in Nutrition

**Figure 7:**
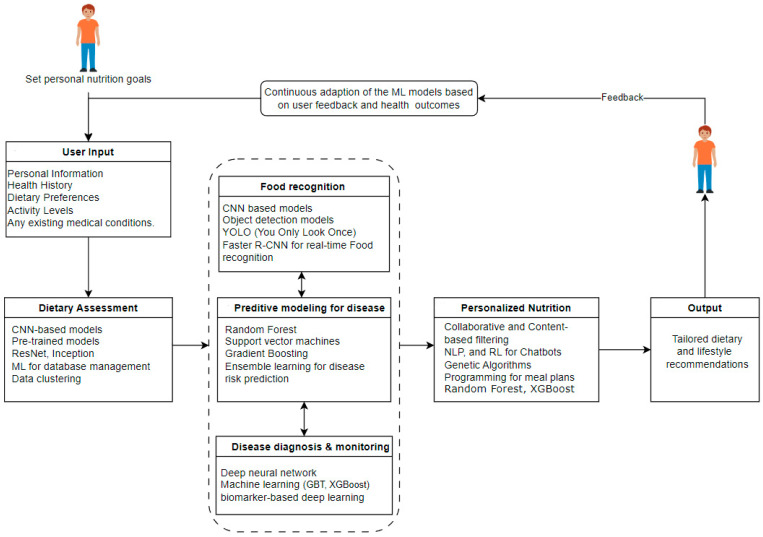
Proposed conceptual framework for AI, ML, and DL applications in nutrition.

The five clusters obtained by grouping the selected research items were used to propose a conceptual framework for applying AI, ML, and DL in Nutrition. We proposed a conceptual framework that utilizes the AI, ML, and DL techniques at each stage of the nutrition process to achieve effective personalized medicine and nutrition goals. The specific methods include but are not limited to those identified during the content analysis process. The research team obtained results mapped to each identified cluster of relevant techniques and instruments indicated in the literature. An adjacent possibility created a flow between the clusters to ensure a complete and effective AI-assisted nutrition system (see Figure 7 below).

The proposed framework for integrating AI into nutrition shows a multi-layered, sophisticated approach to personalized nutrition management. We start with user input, which includes personal information, health history, dietary preferences, activity levels, and existing medical conditions, if applicable. Once this information is obtained, we can proceed into different applications for further steps using different AI technologies to achieve personalized nutrition.

We propose a system where the expected user seeks an effective personalized nutritional recommendation for well-being. The user must provide some basic information to guide their dietary assessment process. Once we have the user’s input, dietary assessment can be carried out to gain insight into foods and drinks consumable over a specified time that corresponds to the required nutrients, energy, and other dietary constituents that fit the user’s goals and abide by nutritional standards. This assignment uses CNN-based algorithms and transfer learning with pre-trained models such as ResNet and Inception for accurate food categorization [32,33,34,35,36,37]. Furthermore, machine learning algorithms can be used to manage databases and data. Data from food diaries, dietary surveys, and wearable devices can be helpful to understand the user’s nutritional habits [53]. Once the assessment is carried out regarding the user’s goal and nutritionist standards, food recognition and tracking applications can assist the users in identifying their food intakes and evaluating their nutritional value quickly.

Vision-based algorithms, including object detection methods, are applied to large-scale food image datasets to detect and classify meals using their images. CNNs and advanced object detection models like YOLO and Faster R-CNN for real-time food recognition can be used in food recognition and tracking [38,39,40,41,54]. With information from food images, wearable cameras, and logging apps, this food recognition and tracking technique aims to provide automated and accurate food consumption tracking, enabling users to monitor and track their dietary patterns effectively. Food recognition is more effective when it follows dietary assessment because it facilitates the user’s decision on food choices and considers user expectations and requirements after the evaluation. Furthermore, patients with specific medical conditions can use AI techniques for disease-predictive modeling, diagnosis, and monitoring [42,43,44,45,46,47,48,49,50,51,52]. The application of AI aims to prevent and control disease development in the human body from a nutritional perspective. AI algorithms can identify patterns associated with disease risk by integrating health records, genetic information, and lifestyle data. Common machine learning state-of-the-art algorithms such as random forest, SVM, gradient boosting, and ensemble learning are employed for disease risk prediction. This facilitates proactive health management, enabling the early detection and prediction of potential health problems. 

Considering an effective dietary assessment and the health risks associated with nutritional intake, AI can facilitate the prediction of personalized recommendations and dietary plans for well-being. Personalized recommendations consider the user’s requirements and nutritional constraints. Customized recommendations are achieved using algorithms like collaborative, content-based, or hybrid methods and deep learning-based approaches such as natural language processing (NLP) and reinforcement learning (RL) for chatbots and next-generation sequencing (NGS) for comprehensive genetic analysis, while random forest (RF) and XGBoost improve personalized nutritional recommendations. Genetic algorithms, particle swarm optimization, and constrained programming are also used for customized meal planning [22,23,24,25,26,27,28,29,30,31,55].

Once all these stages have been implemented, we can deliver personalized nutrition recommendations and observe lifestyle changes through mobile apps, wearable devices, and regular reports. Frequently using AI-based nutrition solutions facilitates behavior change interventions fundamental in promoting sustainable dietary practices. Combining these AI, ML, and DL applications in this domain leverages behavioral insights for personalized interventions, delivering targeted and adaptive strategies for sustaining positive changes over time. A feedback loop can ensure that AI models evolve based on user feedback and updated health data, creating a dynamic and personalized approach to nutrition. This integrated framework aims to achieve personalized nutrition goals, improve health monitoring, and achieve overall health objectives. Each cluster contributes uniquely to a holistic and adaptive approach, using cutting-edge AI technologies to reshape the field of personalized nutrition.

## 5. Discussion

In this literature review, the authors identified five main clusters that represent key areas of application for artificial intelligence (AI), machine learning (ML), and deep learning (DL) in the field of nutrition science.

The first cluster for smart and personalized nutrition covers a wide range of AI, ML, and DL applications in nutrition. This cluster provides the technical potential to deliver smart and personalized nutrition recommendations. Integrating the above-presented technologies with various data sources, including metabolomics, purchase history, and IoT devices, will enable the development of intelligent systems capable of providing accurate nutritional recommendations. Also, diverse techniques such as deep learning, CNNs, and recommendation algorithms will offer a broad-based solution for personalized diet plans for specific health conditions such as chronic kidney disease, hypertension, and many more.

In the dietary assessment cluster, AI techniques are emphasized to improve the efficiency and accuracy of assessing food intake. In this cluster, innovative approaches, such as deep learning with natural language processing, developing mobile AI applications, and creating AI systems capable of estimating calorie and macronutrient content from food images, can be observed. For instance, deep learning can automate dietary assessment by recognizing fake food images and matching food choices with high accuracy. Additionally, mobile AI-based applications are effective in evaluating nutrient intake, indicating the possibility and reliability of integrating AI technologies into different settings in nutrition research. Furthermore, the development and implementation of AI systems and the comparison of various food image recognition platforms emphasize the reliability of these technologies in dietary assessment. All these studies collectively show that AI is a greater option for transforming the methods and tools used for dietary evaluation, thereby reducing manual efforts and introducing more accurate and efficient approaches.

The third cluster on food recognition and tracking shows the use of AI, ML, and DL techniques in combination with other approaches, such as near-infrared hyperspectral imaging, deep learning-based food segmentation, and a combination of CNNs and SVMs for food nutrition content, food image segmentation, and classification for nutritional content assessment. The practical significance of the findings in this cluster is that they show applications of dietary intake monitoring and accurate food recognition and tracking of nutritional intake. The studies in this cluster collectively suggest that AI-based methods can significantly improve food recognition, tracking efficiency, and accuracy, offering new possibilities for food quality control and nutrition monitoring [38,39,40,41]. 

The fourth cluster, “predictive modeling for disease”, clearly shows the role of AI in disease prediction, treatment improvement, and real-time monitoring based on nutritional intake data. Some studies focused on using deep learning models for predicting health conditions, such as overweight/obesity, dyslipidemia, hypertension, and type 2 diabetes mellitus, presenting promising results compared to traditional statistical analysis methods. We also have the development of personalized nutrition recommendation systems, which demonstrate the potential of AI for supporting individuals in managing their nutrition and health. Furthermore, applying deep learning algorithms to predict serum PLP concentration solely based on dietary intake reveals the importance of AI in nutrition assessment and disease prevention. All these studies collectively suggest that AI can reshape clinical nutrition in the future, offering personalized interventions and predictive capabilities for disease prevention and management [42,43,44,45,46,47,48,49].

Lastly, “disease diagnosis and monitoring” shows how AI techniques for studying biomarkers can be used to predict body mass index (BMI) and discover dietary patterns. Based on biochemical profiles, integrating neural networks with metabolomics allows for the evaluation of nutritional biomarkers and the classification of BMI, which is necessary for disease diagnosis and monitoring. Furthermore, some focus can be given to specific health conditions, such as irritable bowel syndrome, which will enable AI to optimize nutrition strategies based on the characteristics of an individual. Additionally, a biomarker-based deep learning system for predicting ideal body weight and classifying body types exists, and it offers a novel approach to personalized dietary recommendations. It is important to note the contribution of understanding the complex relationships between nutrition and health with the help of biomarkers and metabolites. All the studies in this cluster collectively provide a starting point for showing the possibility of AI in enhancing the precision and effectiveness of disease diagnosis and monitoring, thereby providing a solution for personalized nutrition interventions.

After a thorough literature review, the applications of AI, ML, and DL in nutrition research are factual. The areas where these technologies contribute to advancing knowledge can be identified from the five identified clusters, improving methodologies and providing innovative solutions for personalized nutrition, dietary assessment, food recognition and tracking, predictive modeling for disease, and disease diagnosis and monitoring. All these findings collectively show the transformative possibility of AI in reforming the field of nutrition research and promoting personalized approaches to health and well-being.

## 6. Limitations of AI Applications in Nutrition

While reviewing the applications of AI, ML, and DL in nutrition science, the authors identified several limitations that need careful consideration. One prominent challenge in applying AI in nutrition is the quality and availability of data. Many studies included in this review faced limitations regarding the completeness, accuracy, and standardization of dietary and health data [22,34,38,45,50,52,56]. Addressing these issues is crucial for ensuring the reliability and generalizability of AI models in nutritional research. On the other hand, algorithmic bias poses a substantial concern in developing and deploying AI models in nutrition science. Studies within this review acknowledge challenges associated with bias in training data, which may lead to disparities in model predictions across different demographic groups. Ensuring the fair representation and generalization of models across diverse populations remains an ongoing challenge [33,34,38,45,46]. The lack of interpretability and explainability in AI models is a limitation highlighted in several studies. Understanding the rationale behind their predictions becomes challenging as these models become more complex. This limitation raises concerns, particularly in clinical and healthcare settings, where interpretability is essential for gaining trust and facilitating informed decision-making [24,45,50]. Several studies noted limitations in the validation of AI models across diverse populations.

Moreover, the lack of representation of different ethnic, cultural, and socio-economic groups in training datasets may compromise the generalizability of models. Addressing this limitation is essential to ensure that AI applications in nutrition benefit a broad spectrum of individuals. The ethical implications of AI applications in nutrition emerged as a notable challenge [43,47]. Privacy concerns related to collecting and sharing personal health data and the responsible use of AI influencing dietary behaviors require careful consideration. Past studies recognized the need for transparent and ethical frameworks to guide the development and deployment of AI technologies [43]. The scalability of AI applications in nutrition, particularly in healthcare settings, poses a challenge. Integrating AI models into existing healthcare systems and ensuring seamless adoption by healthcare professionals may require overcoming technological, organizational, and regulatory hurdles [24,45]. Effective collaboration between data scientists, nutritionists, healthcare professionals, and policymakers emerged as a challenge. Studies highlighted the importance of fostering interdisciplinary communication to bridge the gap between technical advancements in AI and practical implementation in nutrition science and public health [41,45]. The resource-intensive nature of developing and implementing AI models in nutrition is acknowledged. Studies outlined challenges related to the need for substantial computing power, specialized expertise, and financial resources. Addressing these challenges is essential for promoting the wider accessibility and applicability of AI technologies. In summary, while AI applications in nutrition offer tremendous potential, addressing the identified challenges and limitations is crucial for ensuring AI’s ethical, equitable, and effective integration into this field.

## 7. Further Directions and Research Gaps

Considering the limitations mentioned above regarding the existing literature, the authors identified the following research gaps and further directions for AI’s application in nutrition science.

- Enhancing data quality and standardization: Addressing the challenges related to data quality and standardization emerges as a key focus for future research. Efforts should be directed toward developing robust strategies for improving the completeness, accuracy, and standardization of nutritional and health data. Initiatives promoting open data sharing and collaboration can contribute to creating high-quality datasets for AI model development.
- Mitigating algorithmic bias: Future research should prioritize the development of methods to reduce algorithmic bias in AI models applied to nutrition. Strategies for ensuring diverse and representative training datasets and methodologies for continuous monitoring and adjustment for bias can contribute to developing fair and equitable models across different demographic groups.
- Advancing model interpretability and explainability: To enhance trust and transparency, research is needed to advance the interpretability and explainability of AI models in nutrition. Developing interpretable models and establishing clear communication methods to convey model predictions to end-users, including healthcare professionals and individuals, is crucial for successfully adopting AI technologies in practical settings.
- Validation across diverse populations: Future studies should prioritize validating AI models to ensure generalizability and applicability across different demographic, cultural, and socio-economic groups. Research efforts should emphasize the importance of representative datasets that encompass the diversity of the target population, fostering inclusivity in the development and validation processes.
- Ethical frameworks and privacy protocols: Developing ethical frameworks and robust privacy protocols is imperative to guide the ethical deployment of AI applications in nutrition. Future research should explore ethical considerations, such as consent processes, data ownership, and the responsible use of AI-generated insights, to protect individuals’ rights and privacy.
- Integration into healthcare systems: Efforts should be directed toward overcoming challenges related to the scalability and integration of AI applications into existing healthcare systems. Research should focus on developing strategies to integrate AI technologies into healthcare workflows, ensuring interoperability, and addressing organizational and regulatory barriers to widespread adoption.
- Longitudinal studies and real-world impact: Future research should prioritize longitudinal studies to assess the sustained impact of AI-driven interventions on dietary behaviors and health outcomes. Understanding their long-term effects is essential for evaluating the effectiveness and feasibility of these interventions in real-world settings. Additionally, research should explore the scalability of successful interventions for broader public health impact.
- Interdisciplinary collaboration and communication: Promoting effective multidisciplinary cooperation and communication is vital for successfully developing and implementing AI applications in nutrition. Future research should explore innovative approaches to foster collaboration between data scientists, nutritionists, healthcare professionals, policymakers, and individuals, ensuring a holistic and context-aware approach to AI-driven solutions.
- Resource-efficient approaches: Research should explore resource-efficient approaches to ensure the wider accessibility and applicability of AI technologies in nutrition. This includes developing user-friendly tools, educational resources, and frameworks that empower a broader range of stakeholders, including healthcare professionals and individuals, to leverage AI for improved nutritional outcomes.

Addressing these research directions and gaps will contribute to advancing artificial intelligence applications in nutrition, fostering innovation, ethical deployment, and meaningful impacts on public health.

## 8. Conclusions

This review presents a comprehensive overview of the current landscape of AI, ML, and DL applications in nutrition sciences. Through a synthesis of thorough selected studies, the authors explored the transformative potential of AI in shaping dietary research, analysis, and interventions. The review underscores the breadth of AI applications in nutrition, from dietary assessment to food recognition and tracking, predictive modeling for disease, disease diagnosis and monitoring, and personalized nutrition. From these applications, we observed a paradigm shift in how nutritional research offers innovation tools and gives insights that hold promising potential in improving individual health outcomes and advancing public health initiatives. Though the proposed systems are either in the early stages of development or conceptual phases, and most studies propose concepts, prototypes, or initial experiments with limited mature industrial applications, stakeholders can use them as a guide for enhancing industrial outcomes. This study has uncovered promising achievements, notable challenges, and avenues for future exploration, highlighting the dynamic interplay between technology and nutrition science with significant implications for researchers, practitioners, and policymakers. By offering insights into the achievements and challenges of AI applications in nutrition, it informs the development of evidence-based strategies for leveraging technology to improve dietary behaviors, prevent diseases, and promote overall well-being. The ethical considerations highlighted underscore the need for responsible deployment and user-centric approaches in developing AI-driven interventions.

## Figures and Tables

**Table 1:** Search string characteristics.

Keywords (1)	Keywords (2)	Language	Timeframe	Paper Type
Artificial Intelligence	Diet	---	---	CP OR CR
Machine Learning	Nutrition	---	---	AR
Deep Learning	Food	---	---	RE
Recommendation	Dietary assessment	English	2019–2024	---
**---**	Personalized	---	---	---
**---**	Meal planning	---	---	---
	Healthy eating			

**Table 2:** Selected research records.

Suitability	Records	Records (%)
Highly Suitable	31	77.5
Fairly Suitable	5	12.5
Suitable	4	10
Total	40	100

**Table 3:** Clusters of the identified studies.

Clusters	References	Records	Records (%)
Smart and personalized nutrition	[22,23,24,25,26,27,28,29,30,31]	10	32.3
Dietary assessment	[32,33,34,35,36,37]	6	19.4
Food recognition and tracking	[38,39,40,41]	4	12.9
Predictive modeling for disease	[42,43,44,45,46,47,48,49]	8	25.8
Disease diagnosis and monitoring	[50,51,52,53]	3	9.7

**Table 4:** Summary of the SLR of artificial intelligence, machine learning, and deep learning in nutrition.

No.	Author(s) & Year	Cluster	Category	AI Technology	Proposed Solution	Dataset	Evaluation Metric	Content	Ref. No
1	Kirk et al., 2022	Smart and Personalized Nutrition	ML	RF, XGBoost	Bridge the knowledge gap between AI and nutrition	---	---	A comprehensive guide to integrating ML into nutritional sciences that covers its fundamental concepts, its precision nutrition and metabolomics applications, and a framework for practical applications.	[22]
2	Zhu and Wang 2023	Smart and Personalized Nutrition	ML DL	SVM, NN, NLP, and Vision-Based Method.	Evaluation of AI technologies and nutrition	Genome sequencing data, Vision-based DA, and large-scale recipe datasets	---	Role of AI in enhancing food nutrition and addressing global nutritional challenges and applications such as personalized nutrition recommendations, food manufacturing, and DA.	[23]
3	Van Erp et al., 2021	Smart and Personalized Nutrition	DL	NLP	State-of-the- art and some use cases	---	---	It gives the benefits of applying NLP and AI to analyze recipes, provide personalized recommendations, and aid understanding.	[24]
4	Honda and Nishi 2021	Smart and Personalized Nutrition	ML	---	Food analyzer and recommender.	Purchase history data from supermarkets	---	An approach for household nutrition analysis and personalized food recommendation based on purchase history data	[25]
5	Santhuja et al., 2023	Smart and Personalized Nutrition	ML	SVM	Intelligent, personalized nutrition system	IoT for data collection	Average accuracy of 86%	Intelligent Personalized Nutrition System using real-time data from IoT devices, including smartphones and cameras. SVM is used for accurate nutritional classification	[26]
6	Maurya et al., 2019	Smart and Personalized Nutrition	ML DL	Back propagation, NN, RF, RBF	Kidney diagnosis and diet recommender	UCI ML repository and various hospitals in Mumbai	---	A system using machine learning that predicts the stages of Chronic Kidney Disease and provides a personalized recommended diet based on patient data	[27]
7	Mogaveera et al., 2021	Smart and Personalized Nutrition	ML	Decision Tree (C4.5)	e-Health monitoring system.	USDA food database, UCI CK dataset, Health Calabria Food Database	Accuracy: 91.45%	Designed to improve the health of patients suffering from chronic diseases by providing personalized diet and exercise plans based on their health data and the latest reports	[28]
8	Iwendi et al., 2020	Smart and Personalized Nutrition	ML DL	LR, NB, RNN MLP, GRU, LSTM.	Iomt-assisted patient diet recommendation system	Data of 1000 products and 30 patients collected using the IoT and cloud methods	Accuracy: 93.4% Precision: 85.5% Recall: 89.5% F1 score: 99.0%	It uses DL and ML algorithms to analyze patient data and product information and has developed a personalized recommendation system for patients and dieticians	[29]
9	Sookrah et al., 2019	Smart and Personalized Nutrition	ML	Content-based filtering, MLP	DASH diet recommender	USDA Food Composition Database	Accuracy: 99%.	Developed a DASH diet recommendation system intending to promote healthy eating habits for hypertensive patients in Mauritius based on some factors	[30]
10	Iheanacho and Vincent 2022	Smart and Personalized Nutrition	ML	CNN	Classification, recommendation System.	UEC-FOOD 100 FOOD-101	Accuracy: 85.78	Created a system that classifies and recommends healthy food plans based on local dietary habits in West Africa	[31]
11	Mezgec et al., 2019	Dietary Assessment	DL	NLP	Fake food buffet	An FFB experiment in which 124 participants were invited	Accuracy: Fake: 92.18% Matching: 93%.	DL is used for fake-food image recognition, and NLP is used for food matching and standardization	[32]
12	Folson et al., 2023	Dietary Assessment	AI	---	FRANI	West African food composition, RING nutrient composition	---	FRANI is a mobile application for dietary assessment created with artificial intelligence. A total of 36 adolescent females aged 12–18 were used, and the nutrition intake that FRANI measured was compared	[33]
13	Shi et al., 2024	Dietary Assessment	DL	Faster R-CNN, ResNet	Chinalunchtray-99	ChinaLunchTray-99	Accuracy: 90%	Develop a framework for automatic dietary assessment, including tray meal detection and nutrition estimation for Chinese tray meals	[34]
14	Lu et al., 2020	Dietary Assessment	ML DL	DNN, 3D Reconstruction	goFOOD^TM^	Fast food database	Fsum accuracy: 94.4% Fmin accuracy: 83.9%	Developed an AI-based system called goFOODTM that can estimate a meal’s calorie and macronutrient content by capturing food images with a smartphone	[35]
15	Lo et al., 2019	Dietary Assessment	DL	Point completion network	Novel vision-based DA approach	Yale–CMU–Berkeley object dataset	Accuracy: 95.41%	It uses GAN to synthesize multiple views of the same scene, estimate the volume of food, and propose a system for estimating a person’s food intake from a single camera view of their plate	[36]
16	Van Asbroeck and Matthys 2020	Dietary Assessment	ML DL	MK-SVM, IAS, BoF Models.	---	---	---	Provides a comprehensive comparison of the various food image recognition platforms for dietary assessment	[37]
17	Li et al., 2023	Food Recognition and Tracking	ML DL	NIR-HIS RL.	OptmWave	Near-infrared spectral data of scrambled eggs with tomatoes.	DC: 0.9913 and RMSE of 0.3548.	An approach for estimating food integrated with two neural networks to predict protein content and select wavelengths simultaneously	[38]
18	Siemon et al., 2021	Food Recognition and Tracking	DL	STL, U-Net, DeepLab	Assessment of Chinese tray meals	UNIMIB2016 dataset	(CCE) prediction accuracy of 88.3%	It uses hierarchical clustering to provide a novel sequential transfer learning method to improve the performance of DL-based food segmentation	[39]
19	Sripada et al., 2023	Food Recognition and Tracking	ML DL	CNN SVM	Hybrid model for food recognition and tracking	Customized dataset of 995 images	Precision: 96.5% Recall: 96.5% F1 score: 96.5%	Proposed an approach combining a CNN with an SVM to categorize food items into healthy and unhealthy classes	[40]
20	Limketkai et al., 2021	Food Recognition and Tracking	ML Mobile App	Neural Networks	Predict PPGR	Collected from a cohort of 900 healthy individuals	---	Integration of some digital technologies, including mobile applications, wearable devices, and ML, into clinical nutrition	[41]
21	Salinari et al., 2023	Predictive Modeling for Disease	ML DL	ANN, RF, DT, KNN, SVMs, NLP	Integration apps in the field of nutrition	---	---	The potential of AI for improving the treatment of diseases, prediction of diseases, patient care and medication, and monitoring of patients in real time	[42]
22	Singer et al., 2023	Predictive Modeling for Disease	ML DL	XGBoost LightGBM	Assessment and prediction of clinical events	Clinical data from ICU admission	AUROC: 0.89 AUC: 0.933 AUCPR: 0.970 Sensitivity: 74% Specificity: 84%	How AI enhances screening and assessment, its successful application in identifying malnourished cancer patients, and predicting clinical events in intensive care	[43]
23	Kim et al., 2021	Predictive Modeling for Disease	DL ML	ReLU DNN LRDT	Deep learning model	KNHANES	---	A relationship between nutritional intake and risk of developing overweight/obesity, dyslipidemia, hypertension, and type 2 diabetes mellitus	[44]
24	Mitchell et al., 2021	Predictive Modeling for Disease	ML	Attributable Components Analysis (ACA)	GlucoGoalie	---	Accuracy: 89%	A personalized nutrition recommendation system called GlucoGoalie helps individuals to manage diabetes	[45]
25	Ma et al., 2022	Predictive Modeling for Disease	ML DL	LR MLR DNN	Predicting serum PLP concentration	NHANES 2007–2010	R^2^ 0.47 for DLA 0.18 for the MLR model	A deep learning algorithm that predicts serum pyridoxal 5′-phosphate (PLP) concentration provides valuable insights into the association between dietary patterns and serum PLP	[46]
26	Bond et al., 2023	Predictive Modeling for Disease	ML DL	LR. CNNs	CNN ANN	----	---	Discusses the potential of AI to revolutionize clinical nutrition through personalized interventions, disease prevention, ethical considerations, and AI algorithms to analyze genetics, microbiome, and other factors for customized recommendations and predicting risk	[47]
27	Bhat and Ansari, 2021	Predictive Modeling for Disease	ML DL	NB RF DT	Diet recommendation system	----	Precision: 90% Recall: 82% F-Measure: 86% Accuracy: 90%	The authors emphasize the importance of data analysis in healthcare systems. They use machine learning techniques to predict diabetes and recommend proper diets for diabetic patients	[48]
28	Veerasekhar Reddy et al., 2023	Predictive Modeling for Disease	ML DL	CNNs ANN	E-nurse	Diabetic retinopathy dataset	Average Accuracy: 95%	Created an e-college nurse system focusing on BMI measurements and the early detection of Type-2 diabetes with a personalized health center for students, including diet recommendations	[49]
29	Panagoulias et al., 2021	Disease Diagnosis and Monitoring	ML DL	DNN Biomarkers	BMI classification	---	Accuracy: 75% 10-fold cross-validation: 0.665	Proposed the use of metabolomics to study unique chemical fingerprints left by cellular processes and NN to evaluate nutritional biomarkers, predict BMI, and discover dietary patterns	[50]
30	Panagoulias et al., 2021	Disease Diagnosis and Monitoring	ML DL	GBT XGBoost DNN	Micronutrient compositions	Green Genes database	---	Investigated the therapeutic effect of an AI-based personalized diet on IBS patients, using 34 IBS-M patients and applying an algorithm to optimize personalized nutrition strategies	[51]
31	Karakan et al., 2022	Disease Diagnosis and Monitoring	DL	DNN XGBoost	Personalized nutrition	---	ROC-AUC: 0.964 Accuracy: 91%	A biomarker-based system for personalized nutrition that predicts an individual ideal body weight by classifying the body types into three categories, namely underweight, normal, and obese/overweight	[52]

## Abbreviations

ACA	Affordable Care Act
AI	Artificial Intelligence
ANN	Artificial Neural Network
AR	Article Review
AUC	Area Under The Curve
BMI	Body Mass Index
BoF Models	Bag-of-Features Models
CK	Chronic Kidney
CNN	Convolutional Neural Networks
CP	Conference Papers
CR	Conference Reviews
DA	Data Analysis
DC	Dice Coefficient
DeepLab	Deep Labelling for Semantic Image Segmentation
DL	Deep Learning
DLA	Deep Learning Algorithm
DNN	Deep Neural Network
DT	Decision Tree
Faster R-CNN	Faster Region-based Convolutional Neural Network
FFB	Fake Food Buffet
FRANI	Food Recognition Assistance and Nudging Insights
GBT	Gradient Boosting Tree
GRU	Gated Recurrent Unit
IAS	Image Analysis Software
IBS	Irritable Bowel Syndrome
IBS	SSS- IBS Symptom Severity Score
ICU	Intensive Care Unit
IoT	Internet of Things
KNHANES	Korean National Health and Nutrition Examination Survey
KNN	K-Nearest Neighbors
LightGBM	Light Gradient Boosting Machine
LR	Linear Regression
LRDT	Logistic Regression Decision Tree
LSTM	Long Short-Term Memory
MK-SVM	Multiple Kernel Support Vector Machine
ML	Machine Learning
MLP	Multi-Layer Perceptron
MLR	Multivariable linear regression
NB	Naive Bayes
NIR-HIS	Near-Infrared Hyperspectral Imaging System
NLP	Natural Language Processing
NN	Neural Networks
PPGR	Postprandial Glycemic Response
PRISMA	Preferred Reporting Items for Systematic Reviews And Meta-Analyses
RBF	Radial Basis Function
RE	Review
ReLU	Rectified Linear Unit
ResNet	Residual Neural Network
RF	Random Forest
RL	Reinforcement Learning
RMSE	Root Mean Square Error
RNN	Recurrent Neural Network
ROC	Receiver Operating Characteristics
SLR	Systematic Literature Review
STL	Stochastic Gradient Descent with Zero Training Loss
SVM	Support vector machine
SVMs	Support Vector Machines
UCI	University of California, Irvine
U-Net	U-shaped Convolutional Neural Network
USDA	United States Department of Agriculture
XGBoost	eXtreme Gradient Boosting
